# Sensor-based quantification of items used in the MDS-UPDRS-III scale: repetitive lower-limb movements in healthy human participants

**DOI:** 10.3389/fneur.2025.1685247

**Published:** 2026-01-07

**Authors:** Sven Hunziker, Dorian Vogel, Denise Kalt, Stefanie Feiler, Simone Hemm, Alexander Andrea Tarnutzer

**Affiliations:** 1 Institute for Medical Engineering and Medical Informatics, School of Life Sciences, University of Applied Sciences and Arts Northwestern Switzerland, Muttenz, Switzerland; 2 Dynamics and Statistics of Complex Systems, School of Life Sciences, University of Applied Sciences and Arts Northwestern Switzerland, Muttenz, Switzerland; 3 Neurology, Cantonal Hospital of Baden, Baden, Switzerland; 4 Faculty of Medicine, University of Zurich, Zurich, Switzerland

**Keywords:** Parkinson’s disease, quantitative assessment, toe tapping, leg agility, inertial measurement unit, MDS-UPDRS

## Abstract

**Background:**

The Movement Disorder Society Unified Parkinson’s Disease Rating Scale (MDS-UPDRS) is used as a standardized approach to assess motor function in Parkinson’s disease (PD). This assessment is based on the examiner’s subjective judgement and is therefore variable. While quantitative approaches have been evaluated for upper-limb movements, data is scarce on lower-limb movements. Thus, our aim was to implement and assess a setup to quantify lower-limb movements as defined in the MDS-UPDRS in healthy participants introducing new parameters for smoothness and acceleration patterns.

**Methods:**

Twenty-three participants (age-range = 21–31 years) performed five 20-s trials for both lower-limb movement tasks from the MDS-UPDRS-III, i.e., toe tapping (item 3.7) and leg agility (item 3.8). Foot and leg movements were recorded using four inertial measurement units (two per leg: one mounted on the foot and one on the ankle). Biomarkers such as kinematic parameters (e.g., frequency, angular amplitude, movement smoothness, acceleration-based parameters) were extracted to characterize foot-movement dynamics (dominant vs. non-dominant leg), with statistical analyses including linear mixed-effects models applied to four consecutive, non-overlapping 5-s intervals.

**Results:**

A paired Wilcoxon test showed no significant differences in parameters for toe tapping and leg agility based on leg dominance. For toe tapping, the relationship between frequency and angle displayed non-linearity, with a clear decrease in angle with 17.41° −0.83°/interval*t (t = 1–4 time intervals) and no clear decrease in frequency with 2.75 Hz − 0.02 Hz/interval*t. The median frequency and angle for toe tapping were 2.8 Hz and 16° respectively. The median frequency for leg agility was 2.6 Hz.

**Conclusion:**

Reference values could be determined for all parameters including smoothness and acceleration patterns. The quantitative assessment of two MDS-UPDRS-III items shows that temporal changes and adaptation-mechanisms significantly influenced leg-movement dynamics. Reducing exercise duration to 10 s and implementing a metronome with defined frequency could enhance measurement accuracy and reliability, offering more precise parameters for future applications in PD-patients.

## Introduction

Parkinson’s disease (PD) is one of the most common neurodegenerative diseases of the central nervous system worldwide, which is caused by both genetic and environmental factors and cannot be cured (1–3). Symptoms, however, can be alleviated through physical and occupational therapy, surgical interventions and medication (4, 5). PD-related symptoms occur when dopaminergic nerve cells in the basal ganglia, a core area of the cerebrum, show accelerated degeneration and thus loss-of-function (6). The disease affects 1% of all people aged 60 years or more and can rarely occur before the age of 50 years (2). The impairments caused by PD include both motor and non-motor symptoms, which can affect the patient’s quality of life substantially. Motor symptoms include rigidity, tremor, bradykinesia and postural instability, whereas cognitive decline, REM-sleep behavioral disorders and orthostatic hypotension are amongst the most frequent non-motor symptoms (1, 2).

In the clinical assessment of patients with suspected movement disorders, the evaluation of motor function is essential. Adhering to the pivotal motor signs in PD, the assessment of motor properties such as speed, frequency and regularity of repetitive (alternating) movements, muscle tone, and tremor are recommended. This assessment is standardized according to the Movement Disorder Society Unified Parkinson’s Disease Rating Scale (MDS-UPDRS) (motor score Part III) procedure, and contains also testing of both upper and lower limb movements (7). Specifically, testing of finger and toe tapping as well as alternating arm and leg movements has been included. While the MDS-UPDRS-III scale may demonstrate sufficient interrater reliability amongst movement disorders specialists, it is used much more broadly, and thus, real-life inter-rater variability may be substantially higher (8). This limitation can be overcome with a quantitative and standardized characterization of motor function. More precise and reproducible measurements would be advantageous to assess the effectiveness of medication and other therapies and may allow to facilitate diagnosis and earlier detection of disease progression.

Extensive research has been carried out in the field of arm movement analyses. Smartphone-based systems (9), inertial measurement units (IMU) (10), light diodes (11), optical tracking systems (12) or force measuring resistors (12) have been used to obtain a quantitative assessment of upper limb movements. The use of IMUs in gait analyses of PD-patients has also been well researched and is often applied (13–16). In contrast, the existing literature investigating and characterizing quantitatively repetitive or alternating lower limb movements in PD-patients using different sensors and in comparison, to healthy participants is scarce. In a single study, motor exercises included in the MDS-UPDRS-III scale using inertial measurement units, including toe tapping (TT) (item 3.7) and leg agility (LA) (item 3.8) were investigated. Each exercise was performed for 10 s, with comparisons made between PD-patients and age-matched healthy participants (HPs) using one IMU sensor fixed on each foot (14). This study aimed to classify movement patterns in HPs and PD-patients and to provide objective parameters to investigate differences between left and right legs using machine learning. Notably, the best classification performance (accuracy = 1.00) was achieved when using a linear or Gaussian Support Vector Machine (SVM) trained on combined data from both the upper (96 features) and lower limbs (78 features). The main limitation of this study was the lack of normative reference data, which limits clinical validation and generalizability across different stages of PD and Parkinsonism types. Furthermore, the use of only a single foot-mounted sensor limited the diversity of measurable parameters, particularly for leg agility, where features related to movement smoothness, and acceleration behavior are relevant but were not captured. The present study addresses these gaps by introducing a dual-sensor setup (foot and ankle) in HPs, enabling a more comprehensive parameterization of lower-limb motor performance.

The main objective of the present study was to identify additional parameters and to improve quantitative characterization of TT and LA in HPs, using both a foot-mounted and an additional ankle-mounted IMU. The ankle-mounted sensor was included to better capture vertical movement characteristics, particularly relevant for LA, such as acceleration patterns and movement smoothness. It was designed as a proof-of-concept study exploring its feasibility in HPs, with the goal of extending a refined approach to PD-patient populations in future work. Specifically, we aimed to evaluate the behavior of frequency and amplitude over an exercise-duration of 20 s, to analyze the consistency of sensor-derived measurements across HPs and repeated trials and to assess whether leg dominance affects parameters. In this context, consistency refers to the repeatability of calculated parameters within individuals, the variability between individuals and the stability and reliability of the IMU signals over time. This approach is intended to enable the identification of movement patterns that may not be detectable using qualitative clinical assessments alone. We hypothesized that the measurements would show low intra- and inter-subject variability in HPs, thereby providing normative reference values and a reproducible framework for future comparisons with PD-patient data. By establishing a robust quantitative basis for lower limb motor assessment, we aimed to enhance our understanding of leg and foot movement dynamics and to improve diagnostic capabilities.

## Methods

### Participants

Twenty-three healthy, adult human participants (13 women, 10 men) were enrolled for this study. Participants were recruited at the University of Applied Sciences and Arts Northwestern Switzerland (FHNW) in Muttenz through the distribution of flyers among students, employees, and visitors. The average age ± 1 standard deviation (SD) was 24.2 ± 2.7 years (age-range = 21–31 years). The participants were asked about their handedness and their dominant leg. In this study, the dominant leg was defined as the leg with which a football is kicked (17). The ratio of right-handers to left-handers was 20 to 3. The dominant leg matched handedness in 19 of 23 participants (19 right dominant leg; 4 left dominant leg). More detailed information about the participants can be found in the Supplementary Table S1.

Inclusion criteria were: age between 18 and 65 years, ability to provide informed consent, and absence of exclusion criteria. Participants were excluded if they had a history of neurological or systemic disorders affecting cognition or motor function, such as limb palsies, peripheral vestibular deficits, peripheral polyneuropathy, radiculopathy, or leg joint disorders. Additional exclusion criteria were current intake of sedatives, antidepressants, or neuroleptics, disturbances in consciousness, and pregnancy.

This observational study was conducted according to the principles of Helsinki Declaration and approved by the Ethics Committee of Northwestern and Central Switzerland (EKNZ, ID = 2023–01923). Written informed consent was obtained from all patients enrolled in this study, permitting the scientific use of their clinically collected and anonymized data.

### Measurement systems

Four inertial trackers (MTw Awinda, Xsens Technologies B. V., Enschede, Netherlands) were used to measure leg and foot movements. The Xsens MTw Awinda sensor is a wireless miniature IMU consisting of three-dimensional (3D) accelerometers, 3D gyroscopes, 3D magnetometers, a barometer and a thermometer (18). IMUs were attached to both ankles [at the level of the lateral malleolus and both feet using Velcro patches and straps (see Figures A1–A3 for details)]. The individual sensors were placed on the same, pre-defined body parts for each participant. The lacing of the shoe was used to attach the sensor to the back of the foot. Thereby the sensor was placed at the most distal part of the lacing. The IMU-signals were recorded at a sampling rate of 100 Hz. It was assumed in this study that the influence of drift is negligible, as the observed mean drift rate over 2 s across 230 trials was −0.01 °/s with a standard deviation of 0.11 °/s, resulting in an estimated total drift over 20 s of −0.14° ± 2.17° (see Supplementary Figure S1 for details of the estimation), and as the orientation of all motion trackers was reset prior to each individual 20-s trial. The reset involved an alignment procedure in which the participants sat upright in a neutral position with their feet flat and with the lower legs at right angles to the floor, allowing the IMUs to recalibrate their internal reference frames relative to gravity and sensor axes. This means that after each reset, the z-axis of each sensor was aligned with gravity and the direction of the lower leg, while the x-axis was parallel to the floor and pointed along the direction of the foot, ensuring consistent orientation across all participants and sessions. This step redefined the coordinate system for each sensor, minimizing orientation errors and drift over the short trial duration (18).

**Figure 1:**
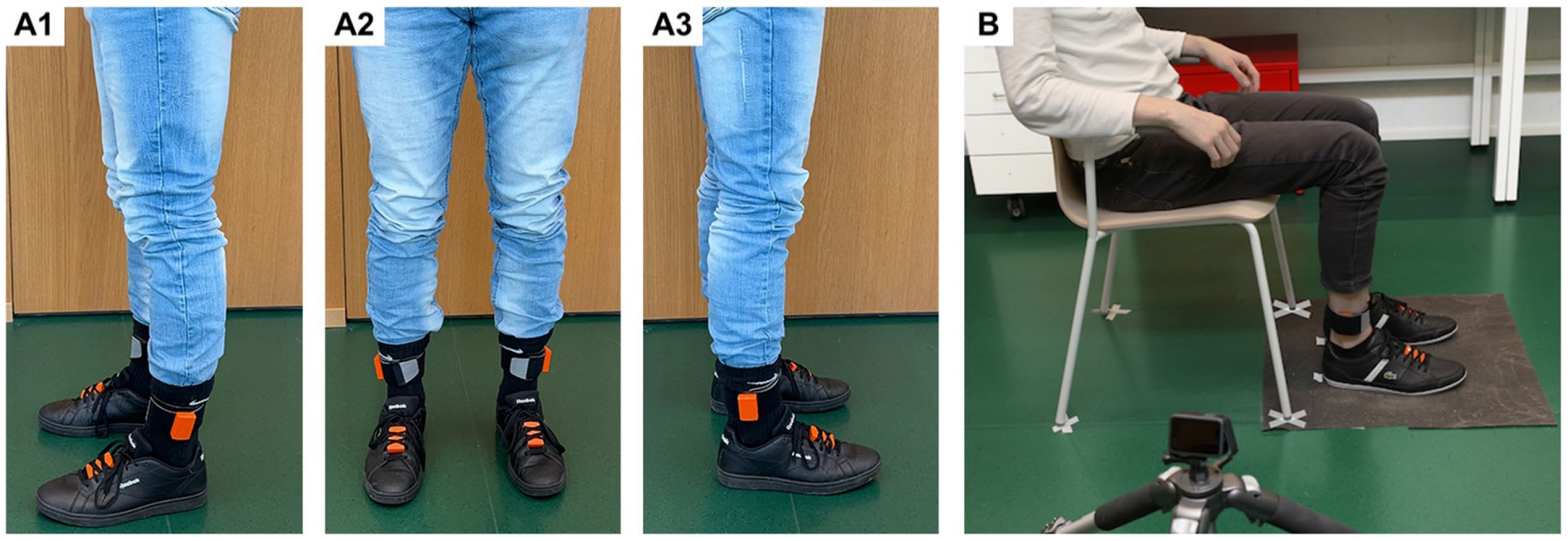
**(A1–A3)** The fully equipped participant during the study had 4 IMUs (MTw Awinda, Xsens Technologies B. V., Enschede, Netherlands) attached to both ankles and feet using Velcro patches and straps. **(B)** The participant was prepared for the examination in the designated neutral position, with tape marks placed at the positions of the chair legs and the ends of the participant’s shoes to ensure consistent positioning throughout the procedure.

In addition, lower leg movements were filmed using a GoPro Hero 10 camera (GoPro Inc., San Mateo CA, USA) to identify possible irregularities such as sensor detachment, incorrect execution of the movement (e.g., incomplete toe taps or leg lifts), unintended pauses or postural deviations that could affect data interpretation.

### Experimental paradigm

The trials were carried out in a laboratory at the FHNW in Muttenz, Switzerland. Each participant sat on a stable chair in neutral position (relaxed, leaning against the backrest, feet parallel, legs at right angles to the floor, arms resting on the armrest) as shown in Figure 1. A non-slip mat was placed on the floor to prevent the participant’s feet from slipping. Each participant performed five 20-s trials for each item (TT item 3.7 and LA item 3.8 of the MDS-UPDRS-III scale) and foot. Data collection was performed in a randomized order for all four items. Before starting the measurements, participants were allowed to get used to the movements and the attached sensors. During this training session, the examiner verified the function of the sensors and their placement and checked and adjusted the participant’s movement speed and dorsiflexion angle or foot height if necessary. Each test began with the participant sitting on the chair in neutral position. Once for each participant’s investigation, a mark was made on the floor behind each end of both shoes with tape to ensure equal positioning of the foot for all repetitions. Test duration was recorded using a timer. For TT, participants were instructed to tap their toes on the floor repetitively with maximal angle and as fast as possible for 20 s. For assessing LA, the participants were instructed to lift the foot and stamp on the floor as high as possible (but at least 20 to 30 cm) and as fast as possible as well over a period of 20 s.

### Data analysis

The raw data recorded with the IMU sensors were processed in Python (v3.9.13) with a Jupyter Notebook (v6.4.12, Project Jupyter, USA). Additional statistical analyses were performed with RStudio Version 2024.9.0.375 (RStudio, Boston, USA) and R Version 4.4.1 (R Core Team, Vienna, Austria). The sensors provided triaxial accelerations, triaxial rotation rates and orientation data in form of Euler angles (roll, pitch, yaw), which were processed to extract kinematic parameters. The orientation was determined onboard by the Xsens proprietary XKF3i Kalman filter algorithm. The sensor fusion algorithm combines data from accelerometers, gyroscopes, and magnetometers in order to provide drift-free 3D orientation estimates. This is achieved by correcting integration drift using gravity and the Earth’s magnetic field (*MTi User Manual*, 2020). The validity of IMU-derived lower leg joint angle measurements during jump-landing and change-of-direction tasks has been confirmed by comparisons with motion capture systems (19).

All sensor data were consolidated into a structured table serving as the basis for feature extraction and analysis. No interpolation was applied to the Xsens signals. The Xsens system performs internal strap-down integration and uses the Awinda protocol, which buffers and retransmits packets to prevent data loss (*MTi User Manual*, 2020). As a result, the exported data stream was complete. No filtering was applied, as the raw signals demonstrated sufficient quality for parameter extraction, with minimal high-frequency noise when compared to a Butterworth low-pass filter (7.5 Hz) (Supplementary Figure S2).

### Segmentation and event detection

The most informative signals were used for segmenting movement and detecting events for parameter extraction. For TT, the data of the raw foot angle in the sagittal plane (pitch) from the foot mounted IMU was used. It is measured orthogonal to the plane in which the tapping motion takes place, providing the clearest representation of the movement. For LA, the vertical acceleration from the ankle mounted IMU was used.

**Figure 2:**
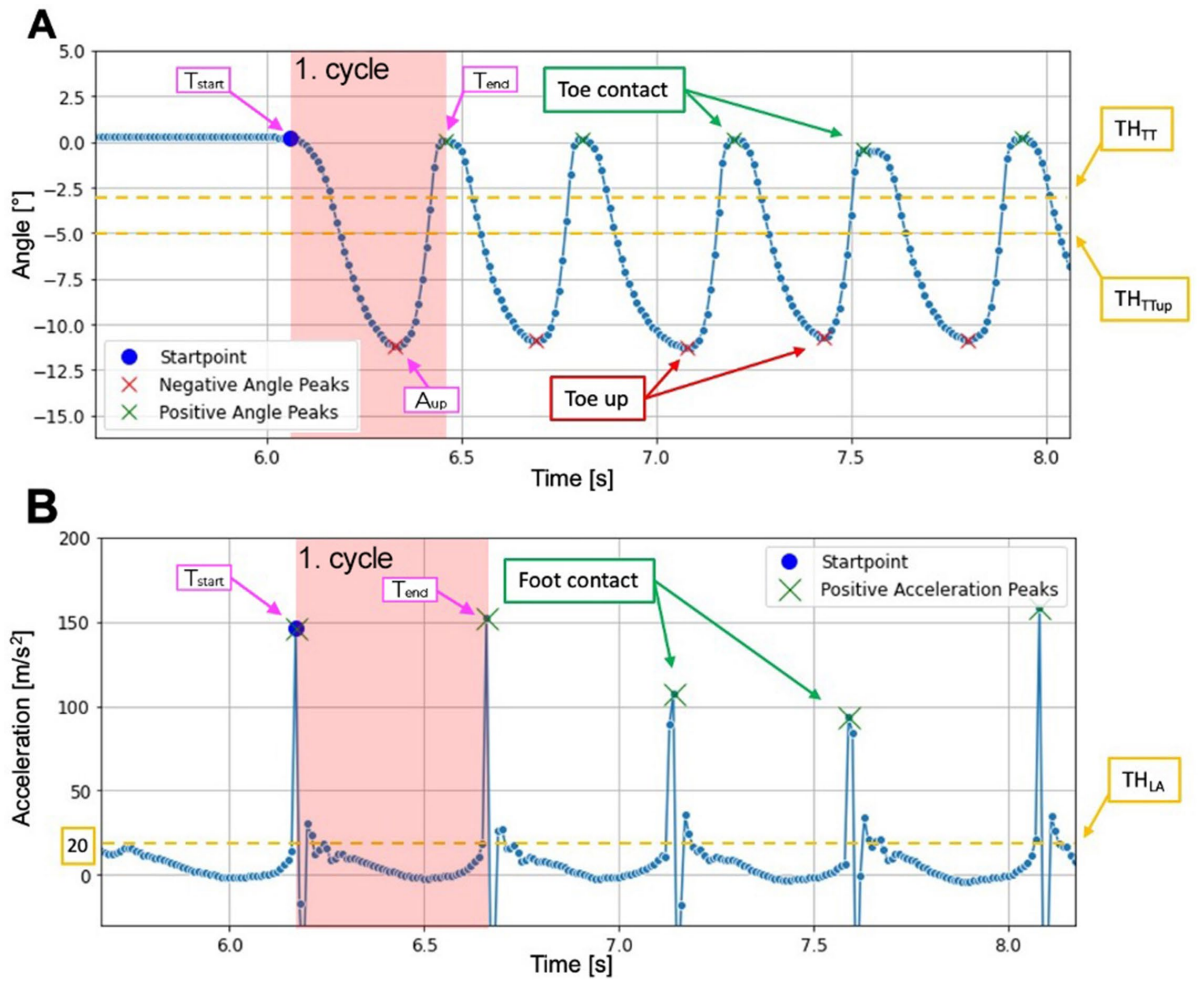
**(A)** Time series of raw foot angle [°] in the sagittal plane (pitch) during toe tapping. Each cycle starts at T_start_ with toe contact, reaches maximum amplitude (A_up_) when the toes are up, and ends at T_end_ with toe contact on the ground. Positive angle peaks (green) indicate toe contact on the ground above the threshold TH_TT_, while negative angle peaks (red) indicate maximum toe elevation below the threshold TH_TTUP_. **(B)** Time series of the raw vertical acceleration data [m/s^2^] during leg agility. Each cycle starts at T_start_ with a positive acceleration peak (green) above the threshold TH_LA_ and ends at T_end_ with the next peak. Positive acceleration peaks indicate foot contact on the ground.

The signals were then automatically segmented by identifying three characteristic time points for each of the two exercises, defining a movement cycle (Figure 2): movement start T_start_, maximum amplitude A_UP_, and movement end T_end_. For the vertical acceleration signal (Figure 2B), no maximum amplitude A_up_ was defined, as this parameter was not observable in this signal.

In addition, thresholds were set based on observed signal behavior to limit the movements and to detect them as a complete cycle: For TT (Figure 2A) a cycle begins when the toes touch the ground (*T_start_*). It can be identified by a positive peak in the pitch signal exceeding the threshold of TT (TH*_TT_*) = −3°. The toes reach their maximum amplitude *A_up_*, when a negative peak in the pitch signal less than *TH_TTup_* = −5° has been detected. Only the heel touches the ground at this position. A cycle ends *T_end_* when the toes touch the ground again. In order to be able to detect the positive peaks, it was ensured that the pitch signal must be at least *TH_TT_* = −3° in neutral position.

For LA (Figure 2B) a cycle starts when the foot touches the ground (*T_start_*). It can be identified by a positive peak of the acceleration exceeding the threshold of LA (*TH_LA_*) = 20 m/s^2^. The foot is 20 to 30 cm above the ground and reaches its maximum amplitude *A_up_*. A cycle ends *T_end_* when the foot is back in contact with the ground and the positive peak of the acceleration is at least *TH_LA_* = 20 m/s^2^.

### Parameter definition

Parameter selection was based on numerous in-house tests. Parameters already presented by Rovini et al. (14) for TT, number of taps, frequency and angle between two consecutive taps, were calculated. Newly developed parameters are dimensionless jerk (DLJ) and logarithmic dimensionless jerk (LDLJ) describing movement smoothness of each movement cycle, a high value representing movement irregularity (20). For LA, the frequency and integral acceleration vector (IAV), previously introduced by Rovini et al. (14), were derived from two consecutive taps. In addition, we calculated the maximum vertical acceleration for each tap. For all parameters, variability was assessed as the relative difference between the maximum and minimum values within each trial. Detailed formula can be found in the appendix (Supplementary material, chapter A.1).

### Parameter extraction and analysis

#### Frequency/angle relationship for toe tapping

TT frequency and angle were determined over the whole 20 s interval for each trial. The relationship between frequency and corresponding angle values of all participants was analyzed over 20 s and for the first 10 s, respectively. It was checked for linear regression (function *OLS* from Python library *statsmodels* v0.13.2). The behavior of angle and frequency over time was analyzed both qualitatively, through visual inspection of color progression of data points over time, and quantitatively, using linear mixed-effects models across four distinct 5-s intervals (function *lmer* from R package *lme4* v1.1.35.5). The Models were fitted using restricted maximum likelihood (REML) estimation. Time was treated as a continuous variable representing the four 5-s intervals, and leg dominance was included as a fixed effect. Participant was modeled as a random effect, allowing both the intercept and slopes for time and dominance to vary across individuals. For angle, the model was specified as Angle ~ Time + (Time + Dominance | Participant), and for frequency as Frequency ~ Time + (Time + Dominance | Participant). These models assumed no fatigue or learning effects as well as the comparability of all repetitions (trials). The model further assumed a linear relationship between the fixed effects and the outcome variables, as well as normally distributed residuals. Homogeneity of variance across leg dominance was assessed using Levene’s test, and multicollinearity among the fixed effects was evaluated with variance inflation factors. Model diagnostics were further supported by visual inspection of residual–fitted value plots and Q–Q plots.

To interpret the strength of the relationship analyzed by linear regression, coefficient of determination (*R*^2^) values were classified according to commonly used guidelines: values between <0.10 were considered negligible, 0.10 to 0.15 weak, 0.16 to 0.48 moderate, 0.49 to 0.79 strong, and 0.80 to 1.00 very strong (21).

### Overview of parameters and leg dominance in the first 10 s

Each trial from every participant was analyzed separately, with parameters calculated for the initial 10 s in a similar way as presented by Rovini et al. (14). Parameter medians were first computed for each individual trial. An overall median and interquartile range (IQR) were then calculated across all trials, separately for the dominant and non-dominant leg, to assess variability. Based on the above defined characteristic time points (i.e., T_START_, T_END_), all parameters were calculated for each movement cycle in both the TT and LA paradigms. Parameter distributions were analyzed for significant differences using the Wilcoxon test (non-normal distribution of the five trials per participant). The relative deviation of the IQR from the median (%IQR) was calculated for each parameter to provide an overview of the relative dispersion of the data. For comparison %IQR was calculated as well for the Rovini’s results (14). To interpret the relative dispersion of the parameters, %IQR values were classified as follows: values below 30% were considered small, 30 to 59% moderate, 60 to 119% high, and 120% or more very high. These thresholds follow a proportional rationale in which each category reflects an approximate doubling of relative spread. Because %IQR is not a standardized metric, no established norms exist. The present classification serves as a structured interpretation tailored to this dataset.

## Results

The test lasted approximately 1 h for each participant. Each of the 23 participants completed five repetitions, resulting in a total of 230 trials for the right and left leg. The data acquisition of the Xsens MTw Awinda sensors was successfully completed for all participants and trials.

### Investigation of the frequency/angle relationship for toe tapping

**Figure 3:**
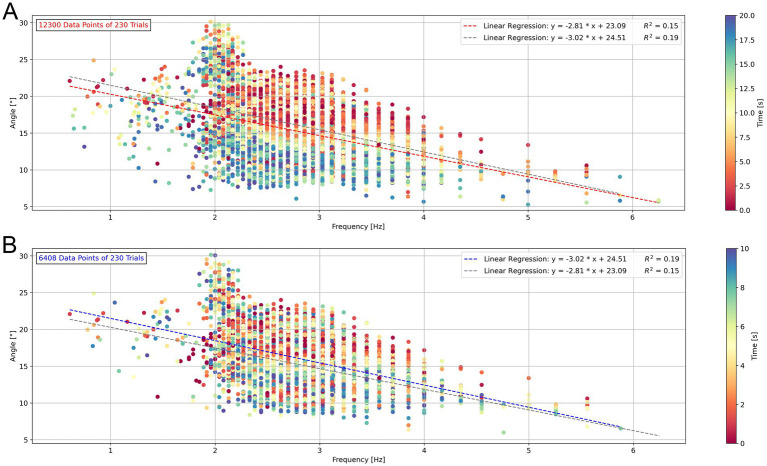
Frequency/angle relationship during toe tapping for all participants for the 20-s test duration **(A)** and for the first 10 s **(B)**. Each tap has a frequency and an angle and is represented as a point. The colors of the points depend on the time in the exercise (A: 0–20s/ B:0–10s). The linear trend line for all data points is shown as a red dashed line. The linear trend line for the data points in the first 10 s is shown as a blue dashed line.

The comparison of the frequency/angle relationship over the entire 20-s duration and the first 10 s of the exercise is presented in Figure 3. Each tap has a given frequency and was plotted against the corresponding angle, represented as individual samples. To visualize changes over time, the color of the data points was adapted depending on the time point of the plotted cycle during the exercise. The red regression line in panel A and the blue regression line in panel B are the regression lines that were calculated in their respective panel with the available samples shown there (A: N = 12,300 vs. B: N = 6,408). Each regression line is reported in the other panel in grey. The color scale between the figures differs, so the Figures 3A,B must be compared with caution.

The frequency/angle relationship for all 12,300 samples over the entire 20-s duration (Figure 3A) showed a significant relationship between the frequency and angle (*p* < 0.001), with a weak correlation (coefficient of determination *R*^2^ = 0.15). The angle clearly decreased during the exercise, indicated by more late time samples (blue) below the red linear trend line. More precisely, the angle decreased linearly across time intervals, as estimated by the linear mixed-effects model, with a fixed-effect regression equation of 17.41°−0.83°/interval*t, where t is the time interval (1 to 4, representing intervals from 0 to 20s in 5 s steps). In contrast, the frequency showed no clear linear trend, with the model estimating a decline of 2.75 Hz − 0.02 Hz/interval*t. This pattern is also reflected in the distribution of angle and frequency values across time intervals, as shown in the box plots (Supplementary Figures S3, S4). All model assumptions were met. Residual–fitted value plots and Q–Q plots showed no deviations from linearity or normality (Supplementary Figure S5), Levene’s test indicated no significant variance differences between leg dominance (*p* = 0.356), and variance inflation factors confirmed the absence of multicollinearity (VIF: Time = 1, Dominance = 1).

The analysis of the frequency/angle relationship in the first 10 s (Figure 3B) showed as well a significant relationship (*p* < 0.001), with a moderate correlation (coefficient of determination *R*^2^ = 0.19), based on 6,408 samples. The sample number outlines, that more than half of the data points originate from the first 10 s of the exercise. While the visual trend in Figure 3B does not show a clearly recognizable decrease in angle over time anymore, statistical analysis using the linear mixed-effects model (Angle ~ Time + (Time + Dominance | Participant)) still indicated a significant linear decline in angle across time intervals.

The angle and frequency analysis according to the dominant and non-dominant leg for each participant showed a linear decrease of the angle and a constant behavior of the frequency in the different time intervals. Additionally, variations in participant behavior across repetitions suggest that fatigue or learning effects are unlikely (Supplementary Figures S6, S7).

### Comparison of parameters in the first 10 s for toe tapping

The comparison of median values between dominant and non-dominant leg showed for TT that the median of taps and frequency as well as TT angle over the first 10 s were in a similar range between dominant and non-dominant legs (Table 1). This focus on the first 10 s was chosen to align with the time duration used in the previous literature for comparable assessments. The DLJ and the LDLJ were slightly increased in the non-dominant leg compared to the dominant leg indicating less smoothness. The IQR was similar for leg dominance for TT, except for a slightly increased IQR in the non-dominant leg in DLJ and LDLJ. Most of the parameters showed a small %IQR of less than 30% in our study (except variability parameters). The DLJ is the only parameter with a very high %IQR of more than 180%. The %IQR tended to be slightly larger for the dominant leg. No significant differences (Wilcoxon, *p* > 0.05) were observed between the dominant and non-dominant legs for all parameters for TT.

**Table 1:** Median, interquartile range (IQR) and associated deviation (%IQR) of parameters for toe tapping and leg agility.

Parameter	Non-dominant leg		Dominant leg		Left leg (14)				Right leg (14)			
	HPs median (IQR)	%IQR	HPs median (IQR)	%IQR	HPs median (IQR)	%IQR	PD median (IQR)	%IQR	HPs median (IQR)	%IQR	PD median (IQR)	%IQR
TT Taps	28.0 (5.0)	17.9	28.0 (6.0)	21.4	33.0 (11.2)	33.9	29.0 (9.0)	31.0	38.5 (9.2)	23.9	31.0 (9.0)	29.0
TT_Freq [Hz]	2.8 (0.5)	17.9	2.8 (0.6)	21.4	3.3 (1.2)	36.4	2.9 (0.9)	31.0	3.9 (1.0)	25.6	3.1 (1.0)	32.3
TT_Freq_Var [%]	28.1 (13.4)	47.7	25.7 (14.4)	56.0	41.3 (25.4)	61.5	48.3 (23.6)	48.9	39.8 (18.1)	45.5	38.7 (22.8)	58.9
TT_Angle [°]	15.8 (4.1)	25.9	16.2 (4.1)	25.3	10.4 (5.3)	51.0	7.7 (5.8)	75.3	7.2 (5.4)	75.0	7.6 (5.4)	71.1
TT_Angle_Var [%]	21.0 (9.8)	46.7	21.8 (9.7)	44.5	60.3 (35.1)	58.2	70.1 (27.4)	39.1	65.0 (23.5)	36.2	64.1 (27.4)	42.7
TT_DLJ	7061.9 (12855.0)	182.0	5782.8 (11697.9)	202.3	–	–	–	–	–	–	–	–
TT_LDLJ	8.9 (1.7)	19.1	8.7 (1.8)	20.7	–	–	–	–	–	–	–	–
TT_LDLJ_Var [%]	35.3 (18.8)	53.3	35.2 (21.5)	61.1	–	–	–	–	–	–	–	–
TT_IAV	–	–	–	–	108.6 (4.2)	3.9	99.0 (17.7)	17.9	111.5 (4.5)	4.0	101.2 (20.7)	20.5
LA_Taps	26.0 (5.0)	19.2	26.0 (6.0)	23.1	–	–	–	–	–	–	–	–
LA_Freq [Hz]	2.6 (0.5)	19.2	2.6 (0.5)	23.1	3.9 (0.7)	17.9	3.6 (0.8)	22.2	4.3 (0.9)	20.9	3.8 (0.8)	21.1
LA_Freq_Var [%]	12.2 (5.1)	41.8	11.4 (6.2)	54.4	–	–	–	–	–	–	–	–
LA_Acc [m/s^2^]	122.7 (32.7)	26.7	124.8 (34.1)	27.3	–	–	–	–	–	–	–	–
LA_Acc_Var [%]	44.9 (16.7)	37.2	45.4 (12.8)	28.2	–	–	–	–	–	–	–	–
LA_IAV	6.1 (1.6)	26.2	6.2 (1.4)	22.6	140.5 (26.4)	18.8	104.5 (11.8)	11.3	141.4 (25.8)	18.2	102.6 (11.2)	10.9
LA_IAV_Var [%]	34.8 (13.1)	37.6	33.2 (11.9)	35.8	–	–	–	–	–	–	–	–
LA_Average Power	–	–	–	–	81.9 (32.4)	39.6	6.1 (16.6)	272.1	82.8 (37.5)	45.3	7.2 (12.5)	173.6
LA_Peak Power	–	–	–	–	111.1 (80.1)	72.1	127.8 (92.5)	72.4	13.2 (28.4)	215.2	13.3 (23.6)	177.4

Data were recorded for both healthy participants (HPs) and Parkinson’s disease (PD) patients and are categorized by dominant and non-dominant leg for this study for the first 10s and left and right leg for the work of Rovini et al. (14). Empty fields represent parameters that were only contained in one study.

The comparison for TT between the study by Rovini et al. (14) and our study showed higher medians with wider IQR’s as well as higher TT frequencies for both legs in HPs and PD-patients compared to our study, as indicated in Table 1. However, the median TT angle was higher in our study compared to Rovini’s study, which reported lower angles in HPs and even lower angles in PD-patients. The parameter values in our study showed lower IQR and %IQR, as well as significantly smaller medians and IQR’s especially for variability parameters compared to Rovini.

### Comparison of parameters in the first 10 seconds for leg agility

The comparison between the dominant and non-dominant leg for LA number of taps and frequency showed a similar median (Table 1). The newly developed acceleration parameter and the IAV parameter were slightly higher in the dominant leg. The IAV parameter was calculated using the same equation, but in different dimensions than in the publication by Rovini et al. (14). The variability parameters demonstrated similar medians for dominant and non-dominant leg. Our study showed similar IQR between the dominant and non-dominant leg in LA. %IQR were less than 30% for most parameters, excluding variability parameters. The %IQR were slightly higher for the dominant leg, but the differences were not significant. The null hypothesis of no significant difference (Wilcoxon, *p* > 0.05) for leg dominance was accepted for all LA parameters.

The values of our parameters have been summarized together with those of Rovini et al. (14) in Table 1 and most of the LA parameters showed smaller medians, IQRs and %IQRs for our study. The median frequency of LA was higher in the published data by Rovini in HPs and slightly higher in PD-patients compared to our data.

## Discussion

The aim of the present study was to identify and evaluate promising approaches for quantitative measurement of repetitive lower leg movements in HPs in the clinical assessment of motor function for PD-patients according to the MDS-UPDRS-III scale. In this work, twice as many inertial sensors were used compared to a previous study (14). We identified a relationship between the tapping frequency and the angle for toe tapping (TT, item 3.7 in the MDS-UPDRS-III scale), showing a linear decrease in the angle and an almost constant behavior for the frequency during a 20 s TT exercise. Furthermore, no significant differences in TT and leg agility (LA, item 3.8) based on the leg dominance were observed.

### Restriction of data collection to 10 s for toe tapping

In the frequency/angle relationship a decrease of angle and frequency would be expected. Figure 3A showed a decrease in the angle over 20 s, which was statistically confirmed by linear regression. Nevertheless, a higher number of samples in the first half of the measurement period compared to the second half indicates a minimal decrease in frequency, which also corresponds to the results of the statistical analysis. The linear regression of all data points also showed a significant relationship between the frequency and angle, which confirms the expectations. Although the participants were instructed to maximize the angle for the entire duration and to perform the movement as quickly as possible, they tended to focus on achieving the maximum angle at a speed they felt comfortable with. As a result of the effort required to reach the maximum angle, it quickly became apparent that most of the participants were unable to maintain it over the full 20 s period. We therefore propose to reduce the duration to 10 s. The reduced trial duration could lead to better performance and more consistent results while providing sufficient data for a detailed analysis. Shorter durations help untrained and especially PD patients to avoid muscle fatigue and enhance focus. Thus, this adjustment may improve the participant’s compliance and reduce performance variability, laying the groundwork for future validation and reliability studies.

### Setting the speed for toe tapping

Due to the observed constant frequency behavior, we suggest that in future studies the frequency be set using a metronome, for example. This would allow a more focused examination of angular motion as the frequency remains constant, minimizing the influence of frequency variations and improving the accuracy of angular analysis. A metronome ensures a standardized speed, improves the comparability of the results and reduces variability in movement speed, leading to a more accurate assessment of motor function. The test protocol is simplified, making it easier to be performed by participants and facilitating its clinical application. Furthermore, it helps to identify deviations from normal movement patterns, thus is useful for the diagnosis and monitoring of PD.

### New parameters for movement characterization

A significant contribution of this study is the introduction of new parameters to enhance the characterization of lower-limb movements, specifically the dimensionless jerk (DLJ) and the vertical acceleration. The DLJ reflects movement smoothness and shows promise as a sensitive marker for detecting subtle motor impairments. Its relevance is supported by the results of the study, which revealed differences in DLJ and LDLJ between dominant and non-dominant legs even in HPs. This suggests the ability to capture asymmetries that are relevant in the context of PD, where motor symptoms are often lateralized. Furthermore, vertical acceleration was introduced as a new parameter specifically for the LA. This measure, derived from the ankle-mounted IMU, provides a direct and isolated view of the vertical movement dynamics during LA. Whilst the diagnostic value remains to be validated in clinical populations in future studies, we predict that vertical acceleration may be substantially reduced in PD-patients due to impaired leg lifting and bradykinesia, thus making it a potentially strong marker for detecting disease-related motor limitations.

### Comparison of the parameters with the existing literature

Across the 23 HPs, the dominant and non-dominant legs showed identical median values for both the number of taps and frequency, with nearly matching interquartile ranges. These findings support the Wilcoxon test results, which revealed no significant differences between sides. This is most likely due to relatively constant parameters with low spread, very precise measurements and problem-free execution without measurement errors. The slightly increased DLJ and LDLJ for the non-dominant leg may reflect greater coordination challenges, which could be relevant in future studies involving PD patients, who often exhibit motor asymmetry (i.e., more pronounced impairment on one side) that necessitates side-to-side comparison. Based on our results, this comparison will not be affected by leg dominance. Importantly, potential effects of leg dominance should be assessed in future studies with larger sample sizes again.

Comparing our study with previous work (14), as shown in Table 1, we observed higher median frequencies in Rovini’s TT (our data – dominant leg: 2.8 Hz (IQR: 0.6) vs. Rovini right leg: 3.9 Hz (1.0)) and significantly smaller achieved median angles (our data – dominant leg: 16.2° (4.1) vs. Rovini right leg: 7.2° (5.4)). We suggest prioritizing a slower movement, but with the maximum angle. Neglecting the angle and the resulting increase in speed may facilitate muscle cramps, speed-related errors and therefore unfavorable measurement results. Instead, the participants performing the exercise should focus on pushing the amplitude of foot/leg movements as much as they can, instead of pushing speed and thus the influence of muscular problems or involuntary movements is expected to be minimized. Rovini’s larger spread in IQR and the variability parameters may be due to such speed-related errors. Another reason for Rovini’s higher IQR could be the participants’ age and the heterogeneity and disease related issues within the PD patients. Specifically, in their study HPs were about 40 years older than in our study.

The median frequencies for LA were higher in Rovini’s study (our data – dominant leg: 2.6 Hz (0.5) vs. Rovini right leg: 4.3 Hz (0.9)), probably due to differences in movement execution. In our study, young HPs lifted their leg 20 to 30 cm, whereas for Rovini’s study the lift height is not known. The dominant leg, which is used more frequently in daily activities, typically exhibits greater muscle strength. This could be the reason for the slightly higher acceleration and integral acceleration vector for the dominant leg.

The small %IQR of the parameters in our study in Table 1 can be explained by the homogeneity in age and the fitness of the participants. The very high %IQR for the dimensionless Jerk in TT is due to high number of outliers in the millions. In addition, the same information can effectively be represented with the logarithmic dimensionless Jerk. The slightly higher %IQR for the dominant leg may reflect greater differences in mobility or force development for the dominant leg.

The major differences between our results and Rovini’s study can be explained by the non-identical measurement set-up, the non-identical movement sequence and by the fact that the HPs included in our study were on average 40 years younger. While any comparison of the results based on this age difference must be made with caution, the relevance of our findings is highlighted by establishing reference values for young HPs. We propose to include reference values for many different age groups in order to better investigate the dynamics and behavior of repetitive foot movements. In consequence, the significant variation in age groups likely impacts fitness and mobility, which should be carefully considered in the analysis. Reducing the recording duration to 10 s and setting a constant speed for TT and LA could improve recording quality. However, the response of PD-patients to these exercises remains uncertain and requires further investigation. Due to additional IMUs attached to the ankle, new parameters have been developed to improve the characterization of LA. Nevertheless, only a few parameters of LA in the present study overlap with those of Rovini, limiting the comparison and quantitative evaluation of this test paradigm. Additionally, the analysis of frequency and amplitude for the LA item was not feasible due to the lack of amplitude data, which restricts its value when compared to the TT paradigm.

### Limitations

This study has several methodological limitations that should be considered when interpreting the results. Firstly, the study includes only young HPs, which limits the generalizability of the findings to the clinical PD population, which is typically characterized by older age and motor impairments. Results provide first reference values, but the next step would be to establish similar ones for both elderly HPs and PD patients. Secondly, while the current trial duration is 20 s, with a proposed reduction to 10 s per trial, the effect of this change on measurement consistency and participant fatigue in PD patients remains untested. Even so, it is highly likely that PD patients have more difficulties performing the tasks and will get tired earlier than the young HPs. Thirdly, no comparison to a gold standard (e.g., motion capture systems) was performed, which prevents formal validation of accuracy. Nevertheless, as mentioned above, Nijmeijer et al. demonstrated that IMU-based lower limb joint angle measurements correlate well with motion capture data during dynamic tasks, supporting the general validity of this approach (19). Finally, one limitation of the present study lies in the relatively low R^2^ values observed in the regression analyses. These values indicate that only a modest proportion of the variance in angle and frequency could be explained by the models. It suggests substantial variability in the data, likely reflecting natural inter-individual differences and fluctuations in motor behavior, even within a healthy population. Taken together, these limitations suggest that while the observed consistency in HPs is promising, further studies are required to validate this setup in clinical populations. In such additional studies, the thresholds values chosen empirically for movement segmentation might have to be adapted to PD patients. Moreover, it would be interesting to investigate potential learning effects or fatigue which were not considered in our study.

## Conclusion

Our work introduced reference values for different and partially newly introduced kinematic parameters for TT and LA. It shows a linear decrease of the angle parameter and a constant behavior for the frequency parameter during 20-s TT in young HPs. This observed trend and the fact that sufficient data for analysis is available, supports a reduction of future measurements of this MDS-UPDRS-III item to 10 s. To optimize data consistency, we propose that future studies use a metronome set at a frequency of 2–3 Hz. Although the LA frequency parameter remained relatively constant, the use of standardized footwear is recommended in future studies to ensure even better comparability of results. All participants should wear identical, equally tightly laced shoes, as different footwear introduces varying damping properties, which can directly affect the impact energy transferred to the foot and the measured parameters of vertical acceleration and the integral acceleration vector for LA. In addition, this study introduced new parameters such as the dimensionless jerk for TT, reflecting movement smoothness, and vertical acceleration for LA. Both show promise in improving sensitivity to subtle motor changes, particularly in the context of PD-related motor asymmetries and impaired leg lifting. Our study yielded parameter values with significantly lower variability and spread than found in literature, which may be attributed to the use of more consistent measurement methods, internally refined signal processing steps, and a younger, more homogenous sample of HPs. These findings suggest improved consistency in the collected data. Overall, future analyses should separate movements for the dominant and non-dominant leg to assess general fitness levels and muscular differences. With a more precise characterization of the dynamics of foot movement, more subtle alterations in leg performance will be detectable. This is both crucial for assessing treatment response and disease progression.

## Data Availability

The datasets presented in this article are not readily available because of the requirement of participant’s approval for data sharing as requested by the ethics committee. Requests to access the datasets should be directed to the corresponding author.
