# Supplementary material for: Sensor-based quantification of items used in the MDS-UPDRS-III scale: repetitive lower-limb movements in healthy human participants
Source: Front Neurol. 2026 Jan 7;16:1685247. doi: 10.3389/fneur.2025.1685247 (PMC12819309; doi:10.3389/fneur.2025.1685247)
Supplement: Supplementary file 1 [file Supplementary_file_1.docx]

# Appendix

## Calculation of Biomarkers

### Toe Tapping

The parameters for toe tapping were all computed based on signals of the foot mounted MTw Awinda sensors.

- **Number of Movements:** Describes the number of complete movement cycles.

$$\text{Taps } = \text{number of } \left( T_{end} \right)$$

- **Mean Frequency:** Describes the average frequency for all movement cycles.

$$\text{Freq [Hz]}=\frac{\sum_{j}^{Taps} \left( T_{end}\left( j \right)-T_{start}\left( j \right) \right)^{-1}}{Taps},\quad\text{where j = 1,…,Taps}$$

- **Frequency Variability:** Describes the variability of the frequency for all movement cycles.
- $\text{Freq}\text{\_}\text{Var}\text{ [\%]}=\frac{\text{max(Freq(j))}-\text{min(Freq(j))}}{\text{max(Freq(j))}}\cdot100\%$
- with:

$$\text{Freq(j) [Hz]}=\left( T_{end}\left( j \right)-T_{start}\left( j \right) \right)^{-1},\quad\text{where j = 1,…,Taps}$$

- **Mean Max Angle:** Describes the average maximum angle of dorsiflexion (the maximum angle that the toes reach from the ground) for all movement cycles. The angle from the pitch signal, i.e. a rotation around the y-axis, is represented by the formula symbol β.
- $\text{Angle [°]}=\frac{\sum_{j}^{Taps} \left( \text{β(j)} \right)}{Taps}$
- with:

$$\beta\text{(j) [°]}=\text{max}_{t\in\left[ T_{start}\left( j \right),T_{end}\left( j \right) \right]} \beta\left( t \right),\quad\text{where j = 1,…,Taps}$$

- **Angle Variability:** Describes the variability of the angle for all movement cycles.

$$\text{Angle}\text{\_}\text{Var}\text{ [\%]}=\frac{\text{max(β(j))}-\text{min(β(j))}}{\text{max(β(j))}}\cdot100\%$$

- **Mean dimensionless Jerk:** Describes the average signal smoothness for all movement cycles. Where v(t) is the speed of movement (Balasubramanian et al., 2015).
- $DLJ=\frac{\sum_{j}^{Taps} (-\frac{{(t_{2}-t_{1})}^{5}}{v_{peak}^{2}} \cdot\int_{t_{1}}^{t_{2}} \left| \frac{d^{2}v(t)}{{dt}^{2}} \right| dt)}{Taps-1}, where j = 1,...,Taps$
- with:

$$t_{1} = T_{\mathrm{start}}\left( j \right) \quad\text{ und }\quad t_{2} = T_{\mathrm{end}}\left( j \right)$$

$$v_{peak}=\text{max}_{t\in\left[ t_{1},t_{2} \right]}v\left( t \right)$$

- **Mean logarithmic dimensionless Jerk:** Describes the average signal smoothness for all movement cycles (Balasubramanian et al., 2015).

$$LDLJ = - ln|DLJ |$$

- **logarithmic dimensionless Jerk Variability:** Describes the variability of the signal smoothness for all movement cycles.

$$\text{LDLJ}\text{\_}\text{Var}\text{ [\%]}=\frac{\text{max(LDLJ(j))}-\text{min(LDLJ(j))}}{\text{max(LDLJ(j))}}\cdot100\%,\quad\text{where j = 1,…,Taps}$$

### Leg Agility

The parameters for leg agility were all computed based on signals of the ankle mounted MTw Awinda sensors.

- **Number of Movements:** Describes the number of complete movement cycles.

$$\text{Taps }=\text{number of }\left( T_{end} \right)+1$$

- **Mean Frequency:** Describes the average frequency for all movement cycles.

$$\text{Freq [Hz]}=\frac{\sum_{j}^{Taps-1} \left( T_{end}\left( j \right)-T_{start}\left( j \right) \right)^{-1}}{Taps-1},\quad\text{where j = 1,…,Taps}$$

- **Frequency Variability:** Describes the variability of the frequency for all movement cycles.
- $\text{Freq}\text{\_}\text{Var}\text{ [\%]}=\frac{\text{max(Freq(j))}-\text{min(Freq(j))}}{\text{max(Freq(j))}}\cdot100\%$
- with:

$$\text{Freq(j) [Hz]}=\left( T_{end}\left( j \right)-T_{start}\left( j \right) \right)^{-1},\quad\text{where j = 1,…,Taps-1}$$

- **Mean Max Vertical Acceleration:** Describes the average maximum acceleration when the foot strikes the ground for all movement cycles. The acceleration is represented by the formula symbol a. Only the peak values are considered.
- $\text{Acc [m}\text{/}s^{2}\text{]}=\frac{\sum_{j}^{Taps} \left( \text{a(j)} \right)}{Taps}$
- with:

$$\text{a(j) [m}\text{/}s^{2}\text{]}=\text{max}_{t\in\left[ T_{start}\left( j \right),T_{end}\left( j \right) \right]} a\left( t \right),\quad\text{where j = 1,…,Taps}$$

- **Vertical Acceleration Variability:** Describes the variability of acceleration for all movement cycles.

$$\text{Acc}\text{\_}\text{Var}\text{ [\%]}=\frac{\text{max(a(j))}-\text{min(a(j))}}{\text{max(a(j))}}\cdot100\%$$

- **Mean Integral Acceleration Vector:** Describes the average integrated acceleration vector related to the estimated energy expenditure (Cavallo et al., 2006).

$$IAV=\int_{T_{start}\left( j \right)}^{T_{end}\left( j \right)} \sqrt{a_{x}^{2}+a_{y}^{2}+a_{z}^{2}}dt,\quad\text{where j = 1,…,Taps-1}$$

- **Integral Acceleration Vector Variability:** Describes the variability of the integrated acceleration vector for all motion cycles.

$$\text{IAV}\text{\_}\text{Var}\text{ [\%]}=\frac{\text{max(IAV(j))}-\text{min(IAV(j))}}{\text{max(IAV(j))}}\cdot100\%$$

## Participant Information

Detailed information on the participants is listed in the Table 4. Age, gender, handedness and dominant leg were recorded for each participant. The dominant leg differs from the handedness only in the case of participant P.16.

**Table S1**: Participant information with age, gender, handedness and dominant leg.

| **Subject** | **Age** | **Gender** | **Handedness** | **Dominant Leg** |
| --- | --- | --- | --- | --- |
| P.01 | 22 | Female | Right | Right |
| P.02 | 23 | Female | Left | Left |
| P.03 | 23 | Female | Right | Right |
| P.04 | 25 | Male | Left | Left |
| P.05 | 23 | Female | Right | Right |
| P.06 | 25 | Male | Left | Left |
| P.07 | 23 | Female | Right | Right |
| P.08 | 24 | Male | Right | Right |
| P.09 | 26 | Male | Right | Right |
| P.10 | 27 | Female | Right | Right |
| P.11 | 25 | Male | Right | Right |
| P.12 | 23 | Male | Right | Right |
| P.13 | 21 | Female | Right | Right |
| P.14 | 22 | Female | Right | Right |
| P.15 | 31 | Male | Right | Right |
| P.16 | 26 | Male | **Right** | **Left** |
| P.17 | 31 | Male | Right | Right |
| P.18 | 25 | Female | Right | Right |
| P.19 | 22 | Female | Right | Right |
| P.20 | 24 | Female | Right | Right |
| P.21 | 21 | Male | Right | Right |
| P.22 | 22 | Female | Right | Right |
| P.23 | 23 | Female | Right | Right |

## Drift Estimation of Xsens MTw Awinda IMUs


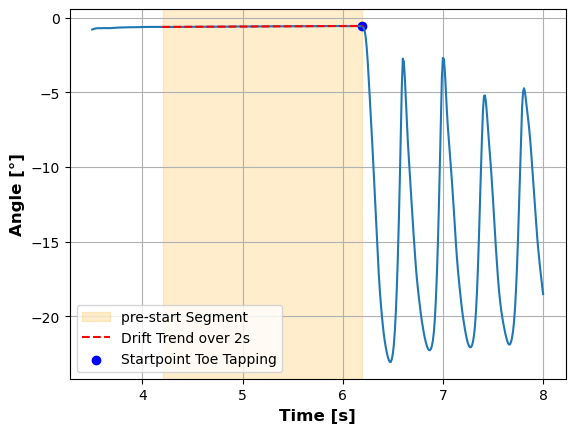


**Figure S1:** Representative example of sensor drift estimation of a raw pitch angle over time for a randomly selected trial of a participant. The shaded orange area highlights the pre-start segment used for drift calculation. A linear regression line (red dashed) fitted to this segment represents the estimated drift rate, and the blue marker indicates the start of the toe-tapping trial. Across 230 trials, the drift per second had a mean of -0.01 °/s with a standard deviation of 0.11 °/s, resulting in an estimated total drift over 20 s of -0.14 ° ± 2.17 °.

## The Comparison of Raw and Filtered IMU Signals


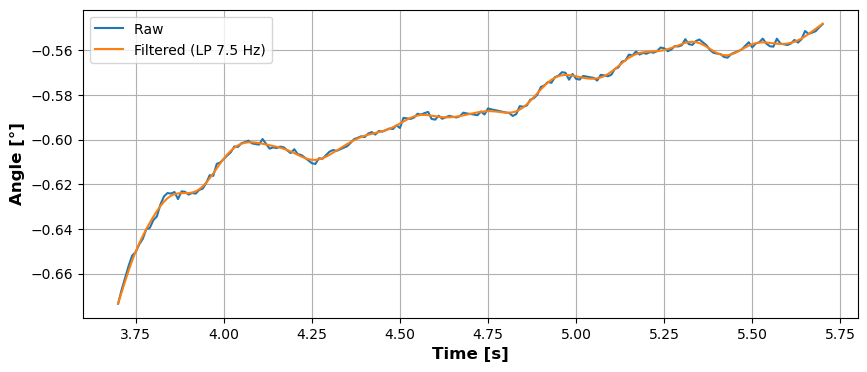


**Figure S2:** Comparison of raw and low-pass filtered pitch angle during a 2-second window before toe-tapping start point for a randomly selected trial of a participant. The raw signal is shown in blue, and the filtered signal (Butterworth low-pass, 7.5 Hz cutoff) is shown in orange, illustrating negligible high-frequency noise and confirming the clean nature of the recorded signal.

## Distribution of the Angle and Frequency in different Time Intervals


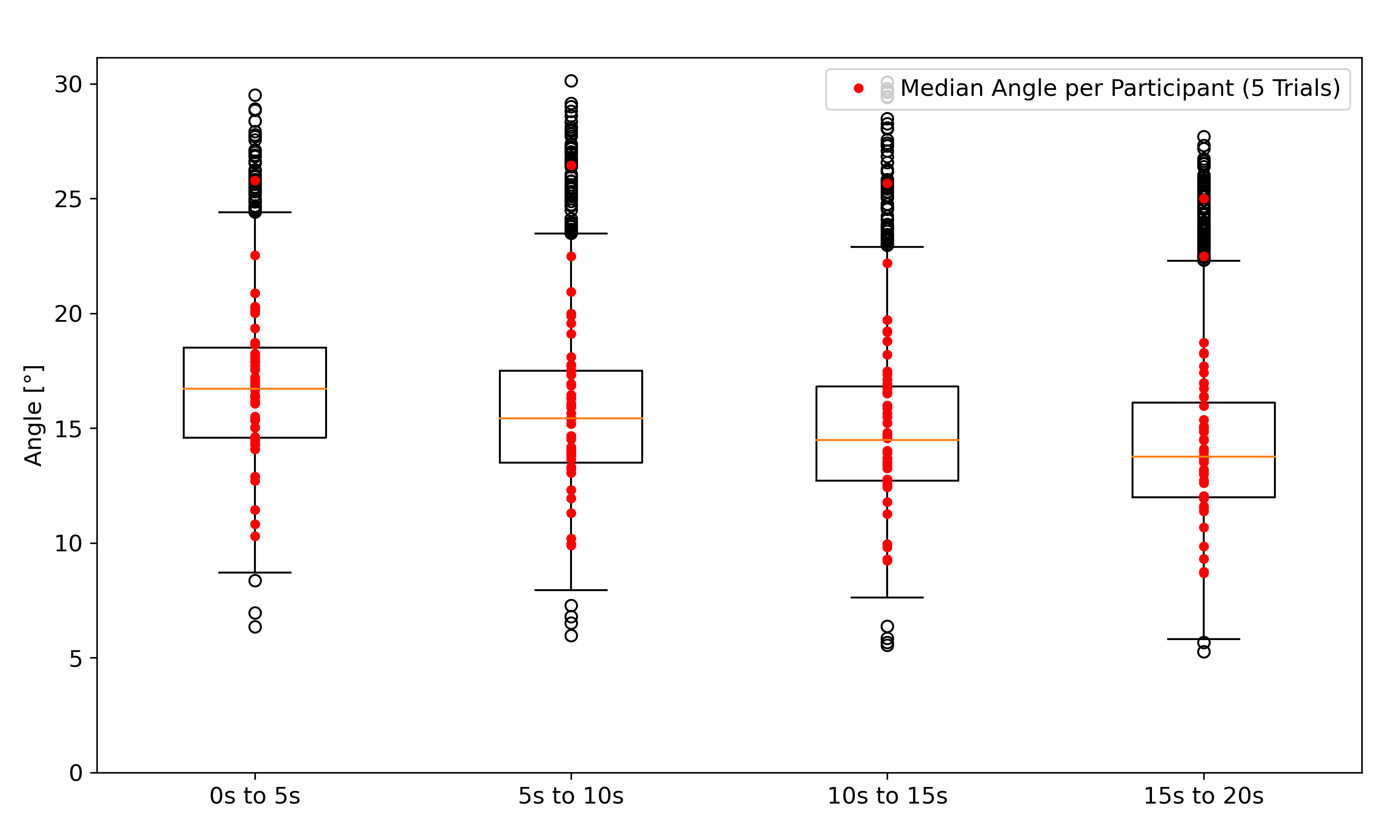


**Figure S3**: Boxplot of the maximum foot angle [°] in the sagittal plane (pitch) across four 5-second intervals (0-5s, 5-10s, 10-15s and 15-20s) during toe tapping. Red dots indicate the median angle per participant, calculated across five trials. Each black circle represents an outlier.


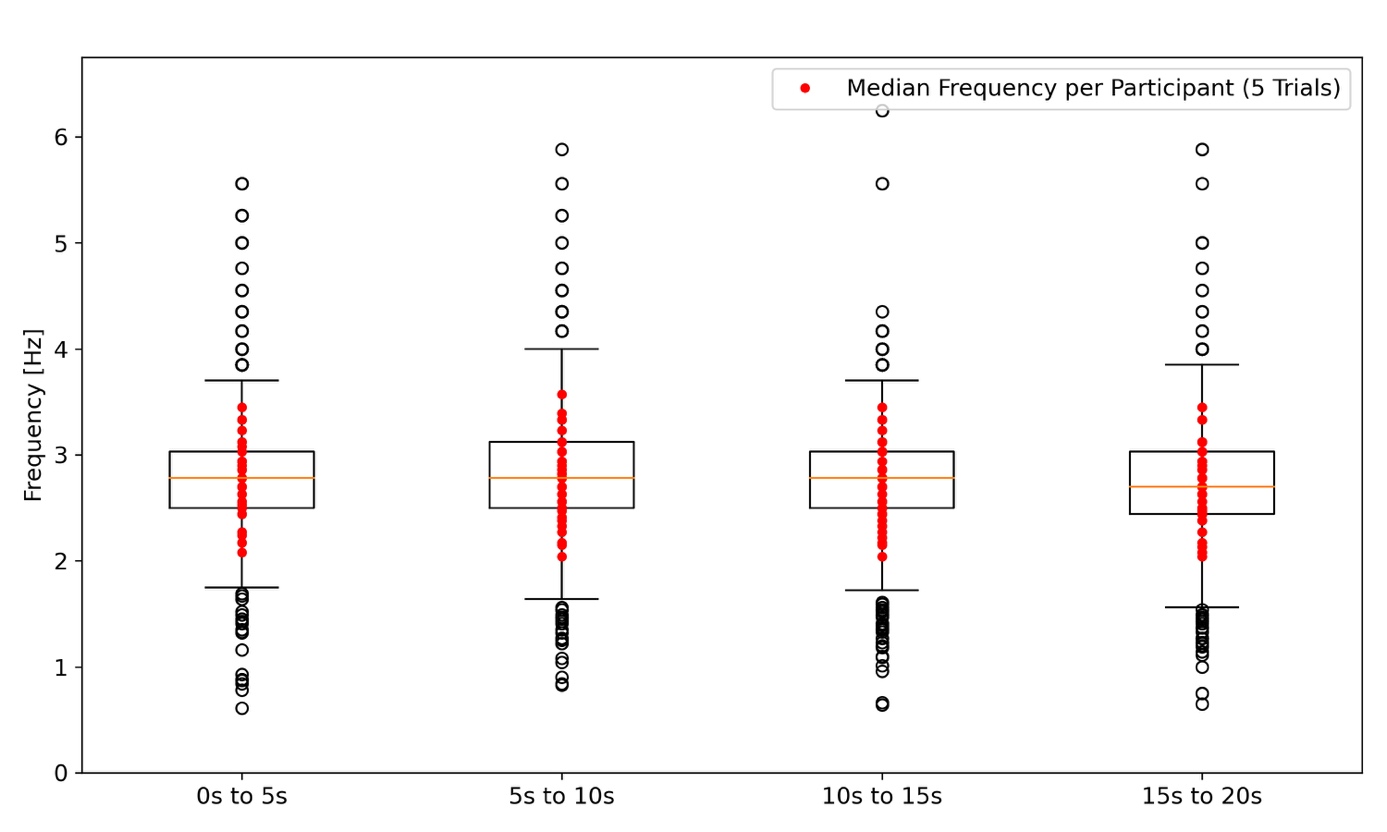


**Figure S4**: Boxplot of the maximum frequency [Hz] across four 5-second intervals (0-5s, 5-10s, 10-15s and 15-20s) during toe tapping. Red dots indicate the median frequency per participant, calculated across five trials. Each black circle represents an outlier.

## Model Diagnostics for Linear Mixed-Effects Models


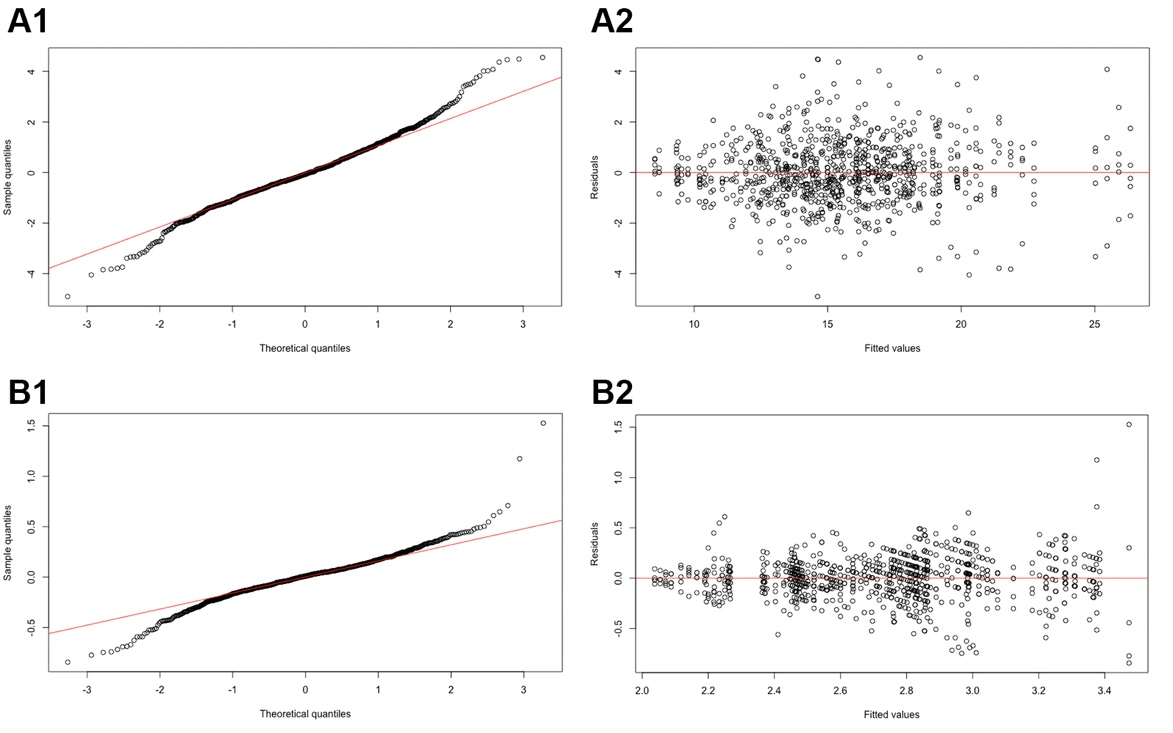


**Figure S5**: Diagnostic plots for the linear mixed-effects models of toe-tapping data. Panels A1 and A2 show the residuals versus fitted values and Q–Q plot for the Angle model, respectively, while panels B1 and B2 show the same for the Frequency model. Both linear mixed-effects models met assumptions of linearity, homoscedasticity, and normality of residuals.

## Angle and Frequency Measurements in different Time Intervals and the Influence of Dominance and Repetition


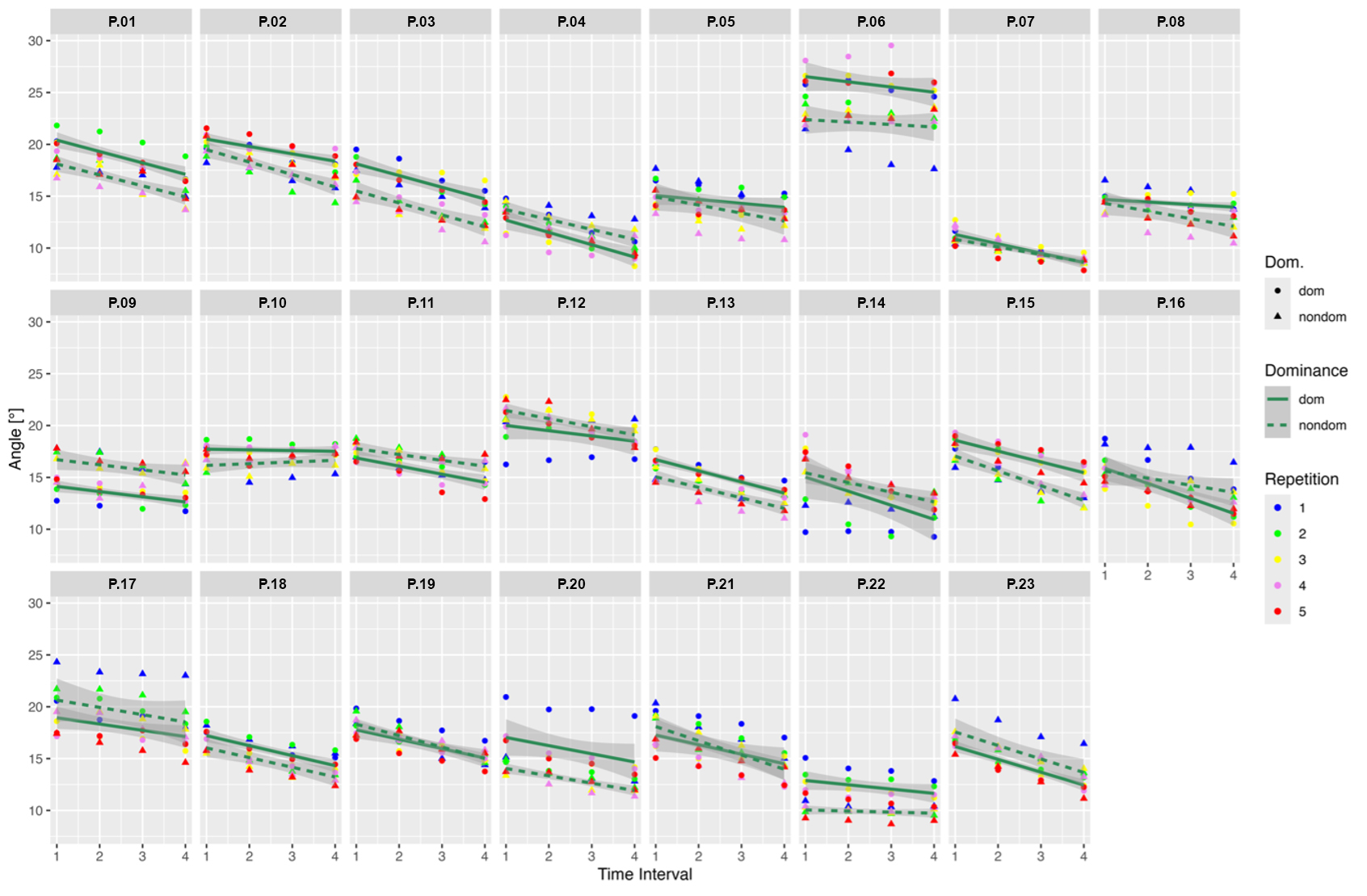


**Figure S6**: Individual participant plots (P.01–P.23) showing the maximum foot angle [°] in the sagittal plane (pitch) across four time intervals (1: 0–5s, 2: 5–10s, 3: 10–15s, 4: 15–20s) during toe tapping. Each dot represents a median value from a single trial, colored by repetition (1–5). Circles indicate dominant leg, and triangles indicate non-dominant leg. Solid and dashed lines represent fitted linear trends for dominant and non-dominant legs, respectively, with shaded areas showing 95% confidence intervals.


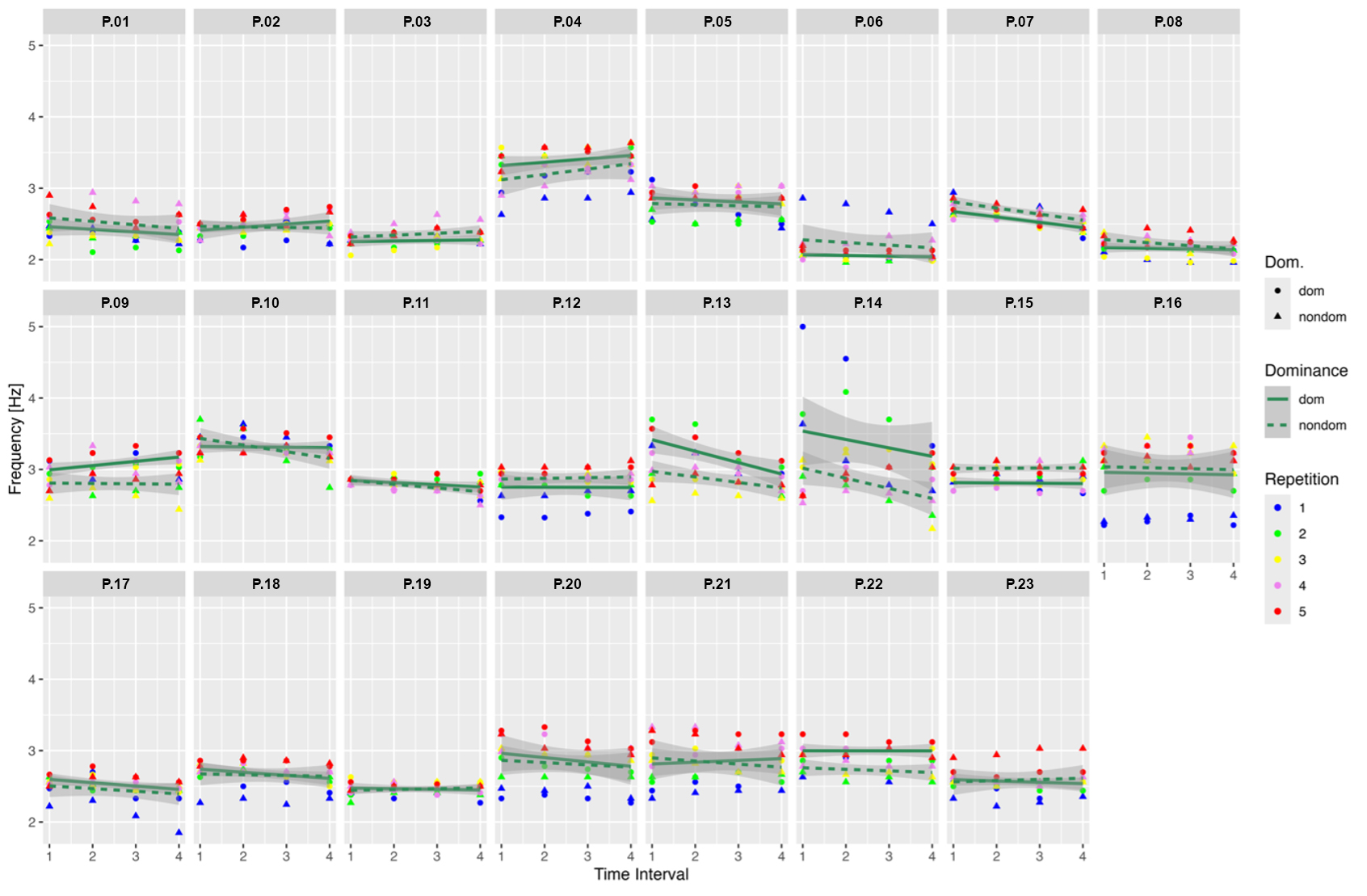


**Figure S7**: Individual participant plots (P.01–P.23) showing the maximum frequency [Hz] across four time intervals (1: 0–5s, 2: 5–10s, 3: 10–15s, 4: 15–20s) during toe tapping. Each dot represents a median value from a single trial, colored by repetition (1–5). Circles indicate dominant leg, and triangles indicate non-dominant leg. Solid and dashed lines represent fitted linear trends for dominant and non-dominant legs, respectively, with shaded areas showing 95% confidence intervals.

## References

Balasubramanian, S., Melendez-Calderon, A., Roby-Brami, A., Burdet, E., 2015. On the analysis of movement smoothness. J Neuroeng Rehabil 12, 112. https://doi.org/10.1186/s12984-015-0090-9

Cavallo, F., Megali, G., Siniaglia, S., Tonet, O., Dario, P., 2006. A biomechanical analysis of surgeon’s gesture in a laparoscopic virtual scenario [WWW Document]. URL https://www.iris.sssup.it/handle/11382/405249?mode=simple (accessed 6.3.24).
